# The Atrial Fibrillation Better Care (ABC) Pathway and Clinical Outcomes in Patients with Atrial Fibrillation: the Prospective Murcia AF Project Phase II Cohort

**DOI:** 10.1007/s11606-022-07567-5

**Published:** 2022-04-11

**Authors:** José Miguel Rivera-Caravaca, Vanessa Roldán, Lorena Martínez-Montesinos, Vicente Vicente, Gregory Y.H. Lip, Francisco Marín

**Affiliations:** 1 Department of Cardiology, Hospital Clínico Universitario Virgen de la Arrixaca, University of Murcia, Instituto Murciano de Investigación Biosanitaria (IMIB-Arrixaca), CIBERCV, Murcia, Spain; 2 Liverpool Centre for Cardiovascular Science, University of Liverpool and Liverpool Heart and Chest Hospital, Liverpool, UK; 3 Department of Hematology and Clinical Oncology, Hospital General Universitario Morales Meseguer, University of Murcia, Instituto Murciano de Investigación Biosanitaria (IMIB-Arrixaca), Murcia, Spain; 4 Department of Clinical Medicine, Aalborg Thrombosis Research Unit, Aalborg University, Aalborg, Denmark

**Keywords:** Atrial fibrillation, ABC pathway, Integrated care, Mortality, MACE

## Abstract

****Background**:**

The Atrial fibrillation Better Care (ABC) pathway was proposed for a more holistic or integrated care approach to atrial fibrillation (AF) management. We investigated whether adherence with the ABC pathway reduced the risk of adverse clinical outcomes in real-world AF patients starting vitamin K antagonist (VKAs) therapy.

****Methods**:**

Prospective cohort study including AF outpatients starting VKA therapy from July 2016 to June 2018. Patients were considered as adherent if all ABC pathway criteria (A: Avoid stroke; B: Better symptom control; and C: Cardiovascular risk factors/comorbidities management) were fulfilled. The primary endpoints were all-cause mortality, net clinical outcomes (NCOs), major adverse cardiovascular events (MACE), and composite thrombotic/thromboembolic events at 2 years.

****Results**:**

We enrolled 1045 patients (51.6% female; median age 77 [70–83] years). Of these, 63.0% (658) were adherent to the ABC pathway and 37% (387) were considered non-adherent. Compared to non-adherent patients, those who were ABC adherent had lower event rates for all-cause mortality (13.76 vs. 6.56; *p*<0.001), NCOs (19.65 vs*.* 11.94; *p*<0.001), and MACE (11.88 vs. 7.75; *p*=0.006) during the follow-up. Adjusted Cox regression analyses demonstrated that the ABC pathway adherent care reduced the risks of all-cause mortality (aHR 0.57, 95% CI 0.42–0.78), NCOs (aHR 0.72, 95% CI 0.56–0.92), and cardiovascular mortality (aHR 0.54, 95% CI 0.32–0.90). Event-free survivals for all-cause mortality, NCOs (both log-rank *p*-values <0.001), and MACE (log-rank *p*-value = 0.004) were also higher in ABC pathway adherent patients.

**Conclusions:**

In this real-world prospective cohort of AF patients starting VKA therapy, adherence to the ABC pathway management at baseline significantly reduced the risk of NCOs, all-cause mortality, and cardiovascular death at 2 years.

**Supplementary Information:**

The online version contains supplementary material available at 10.1007/s11606-022-07567-5.

## INTRODUCTION

The management of atrial fibrillation (AF) has evolved during the last decade. The search for a more comprehensive, integrated, and holistic management of AF patients had led to the proposal of the Atrial fibrillation Better Care (ABC) pathway^1^. This simple strategy, which aims to streamline primary and secondary care of patients with AF, is based on three pillars: “A” avoid stroke; “B” better symptom control; and “C” cardiovascular risk factor and other comorbidities management^1^.

Recognizing this need for a more integrated care approach to AF management, the 2020 European Society of Cardiology (ESC) guidelines on the diagnosis and management of AF have promoted use of the ABC pathway as a simplified and concise approach that integrates the care of AF patients across various levels of healthcare professionals and between specialties, as well as facilitating patient engagement^2^. The key message is that the principals of AF management could be as “Easy as ABC….” (3–5).

The ABC pathway has been tested in retrospective post hoc analyses of clinical trial cohorts^6,7^, nationwide claims data^8^, observational cohorts^9,10^, and one prospective cluster randomized trial, the mAFA-II trial^11^. In all studies, there was general consistency that ABC pathway adherent care was associated with a reduction in clinical outcomes, and in the long-term extension cohort from the mAFA-II trial there was good adherence and persistence^12^. Despite the ABC pathway provides a simple decision-making framework to enable consistent equitable care, the proportion of adherent patients is overall suboptimal, and this is associated with a higher risk of major adverse outcomes^13,14^.

Given the relatively limited prospective evidence of the relationship of the ABC pathway and adverse events in AF patients, particularly in those taking oral anticoagulation (OAC) therapy with vitamin K antagonists (VKAs), we aimed to investigate if adherence to this approach at baseline reduced the risk of adverse clinical outcomes in real-world AF patients starting VKA therapy.

## METHODS

A detailed description of the prospective Murcia AF Project Phase II cohort has previously been published^15^. In brief, this is a prospective observational cohort study including outpatients newly diagnosed with AF and naïve for OAC in an anticoagulation clinic of a tertiary hospital (Murcia, Spain), from July 1, 2016, to June 30, 2018. Eligible patients were those who started OAC with VKAs for the first time. Only those patients older than 18 years with documented evidence of AF on ECG and not previously anticoagulated for another reason were included. Patients with prosthetic heart valves, rheumatic mitral valves, or other type of severe valvular AF were excluded. In order to perform a prospective cohort study that reliably reflects the “real-world” clinical practice, no other exclusion criteria were established. At baseline, a complete medical history was obtained by collecting socio-demographic and anthropometric data, comorbidities, concomitant therapies, and results of the most recent lab tests. In addition, stroke (CHA_2_DS_2_-VASc) and bleeding (HAS-BLED) risk scores were calculated.

The study protocol was approved by the Ethics Committee from the University Hospital Morales Meseguer (reference: EST: 20/16) and was carried out in accordance with the ethical standards established in the 1964 Declaration of Helsinki and its subsequent amendments. Informed consent was required for participation in this study.

### Atrial Fibrillation Better Care Pathway Assessment

The ABC pathway was evaluated at baseline according to its original definition, as follows:

- “A” Criterion: A patient would qualify as adherent for this criterion if properly prescribed and treated with an OAC. As all patients were included in the context of starting VKA therapy (which is common practice in Spain, where a trial of VKA is mandated before consideration of direct-acting OACs [DOACs]) and no previous data about the time in therapeutic range (TTR) were available, the “A” criterion was considered fulfilled if VKA was correctly prescribed according to thromboembolic risk (i.e., CHA_2_DS_2_-VASc >1 in males or CHA_2_DS_2_-VASc ≥2 in females).
- “B” Criterion: Defined as the presence of symptoms related to AF, classified by the recommended European Heart Rhythm Association (EHRA) symptom scale. Any patient with an EHRA score of I (no symptoms) or II (mild symptoms not affecting daily life) was considered adherent to this criterion whereas patients with EHRA score of III (severe symptoms) or IV (disabling symptoms) were considered non-adherent. Data on symptoms were collected at baseline.
- “C” Criterion: Defined as the optimal management/medical treatment of the main cardiovascular comorbidities: hypertension, coronary artery disease, peripheral artery disease, heart failure, stroke/transient ischemic attack (TIA), and diabetes mellitus. Optimal medical treatment was defined as follows: (i) for hypertension, we considered controlled blood pressure if <160/90 mmHg was recorded at baseline and treated with appropriate drugs; (ii) for coronary artery disease, treatment with angiotensin-converting enzyme (ACE) inhibitors, beta-blockers, and statins; (iii) for peripheral artery disease, treatment with statins; (iv) for previous stroke/TIA, treatment with statins; (v) for heart failure, we considered treatment with ACE inhibitors/angiotensin receptor blockers and beta-blockers; (vi) for diabetes mellitus, treatment with insulin or oral antidiabetics. To be included as adherent to “C” criterion, all considered risk factors should have been well controlled and/or treated with appropriate cardiovascular preventive drugs.

A patient was considered as fully ABC pathway adherent (“ABC adherent care”) if all the three criteria were fulfilled.

### Follow-up and Clinical Outcomes

Follow-up was performed according to the standard of care at each routine visit to the outpatient anticoagulation clinic or visits for the anticoagulation control. If the patient never attends to these visits, medical records and telephone calls were used to obtain the information needed and vital status, with no specific interventions and no specific visits for study purposes. Follow-up was extended for 2 years in ABC pathway adherent and non-adherent patients, with no difference between both groups. During this period, all adverse events were recorded. Of note, patients lost to follow-up were <1% of the overall sample and thus unlikely to skew results with a sensitivity analysis evaluating them as intention-to-treat.

For the present study, the *primary endpoints* were all-cause mortality, net clinical outcomes (as the composite of major bleeding, ischemic stroke/TIA, and all-cause mortality), major adverse cardiovascular events (MACE, as the composite of fatal/nonfatal myocardial infarction, cardiovascular death, and ischemic stroke/TIA), and composite thrombotic/thromboembolic events (any of the following: myocardial infarction, ischemic stroke/TIA, venous thromboembolism [VTE, including both deep vein thrombosis and pulmonary embolism]). *Secondary outcomes* were the individual outcomes of ischemic stroke, TIA, myocardial infarction, VTE, major bleeding (defined based on 2005 International Society on Thrombosis and Haemostasis (ISTH) criteria (16)), intracranial hemorrhage (ICH), clinically relevant non-major bleeding (CRNMB, according to the 2015 ISTH criteria (17)), and cardiovascular death*.* The investigators identified, confirmed, and recorded all clinical outcomes.

### Statistical Analyses

Continuous variables were expressed as mean ± standard deviation (SD) or median and interquartile range (IQR) as appropriate, while categorical variables were expressed as absolute frequencies and percentages. The Pearson Chi-squared test was used to compare proportions and differences between continuous and categorical variables were assessed using the Mann-Whitney *U* test or the Student *t* test, as appropriate.

Cox proportional hazard regression models were performed to determine the association between the ABC pathway and the primary endpoints. A univariate significance level of 0.05 was required to allow a variable into the multivariate model (SLENTRY = 0.05) and a multivariate significance level of 0.05 was required for a variable to stay in the model (SLSTAY = 0.05). Results were reported as hazard ratio (HR) with 95% confidence interval (CI).

Annual event rates with their Poisson 95% CI were calculated for ABC pathway adherent and non-adherent as the number of adverse clinical outcomes divided by the exposure period in patients-years (PYs), and expressed as number of events per 100 PYs. The difference between two annual event rates and the associated *p*-value was calculated. Annual event rates (i.e., incidence rates) were also compared and reported as incidence rate ratio (IRR). To calculate IRR, the event rates for every endpoint in the non-adherent group (R1) were divided by the event rates for every endpoint in the adherent group (R2). An IRR <1 indicated that the incident rate was lower in the non-adherent group compared to that in the adherent group. An IRR >1 indicated that the incident rate was higher in the non-adherent group compared to that in the adherent group. IRRs = 1 indicated no differences in the incidence rates of both groups. Finally, survival analyses by Kaplan-Meier estimates were performed to assess differences in event-free survival distributions, which were compared using the log-rank test.

A *p*-value <0.05 was accepted as statistically significant. Statistical analyses were performed using SPSS v. 25.0 (SPSS, Inc., Chicago, IL, USA), and MedCalc v. 16.4.3 (MedCalc Software bvba, Ostend, Belgium) for Windows.

## RESULTS

Overall, 1254 AF patients were initially evaluated. Of these, 1064 patients fulfilled inclusion/exclusion criteria and were included, but 14 patients were lost to follow-up giving a final study cohort of 1050 patients. Of these, 1045 (51.6% female; median age 77, IQR 70–83 years) with a median CHA_2_DS_2_-VASc of 4 (IQR 3–5) and HAS-BLED of 2 (IQR 2–3), had complete data for the present analysis. Table 1 gives a summary of baseline clinical characteristics.

**Table 1.** Baseline Clinical Characteristics

	**Overall** ***N*** **= 1045**	**ABC pathway non-adherent** ***N*** **= 387**	**ABC pathway adherent** ***N*** **= 658**	***p*-value**
**Demographic**				
Male sex, *n* (%)	506 (48.4)	196 (50.6)	310 (47.1)	0.270
Age (years), median (IQR)	77 (70–83)	78 (70–84)	76 (70–82)	0.212
BMI (kg/m^2^), median (IQR)	30.0 (26.8–33.3)	30.1 (26.8–33.3)	30.0 (26.8–33.3)	0.833
**Comorbidities,** ***n*** **(%)**				
Hypertension	874 (83.6)	322 (83.2)	522 (83.9)	0.772
Diabetes mellitus	393 (37.6)	224 (57.9)	169 (25.7)	<0.001
Heart failure	261 (25.0)	135 (34.9)	126 (19.1)	<0.001
History of stroke/TIA/thromboembolism	162 (15.5)	70 (18.1)	92 (14.0)	0.077
Renal impairment	197 (18.9)	95 (24.5)	102 (15.5)	<0.001
Coronary artery disease	190 (18.2)	97 (25.1)	93 (14.1)	<0.001
Peripheral artery disease	66 (6.3)	48 (12.4)	18 (2.7)	<0.001
Hypercholesterolemia	608 (58.2)	218 (56.3)	390 (59.3)	0.352
Current smoking habit	157 (15.0)	65 (16.8)	92 (14.0)	0.219
Current alcohol consumption	71 (6.8)	26 (6.7)	45 (6.8)	0.940
History of previous bleeding	173 (16.6)	68 (17.6)	105 (16.0)	0.498
COPD/OSAH	230 (22.0)	92 (23.8)	138 (21.0)	0.291
Hepatic disease	68 (6.5)	25 (6.5)	43 (6.5)	0.962
Concomitant malignant disease	150 (14.4)	55 (14.2)	95 (14.4)	0.920
**Concomitant treatment,** ***n*** **(%)**				
Antiarrhythmics	214 (20.5)	76 (19.6)	138 (21.0)	0.606
Calcium antagonist	317 (30.3)	126 (32.6)	191 (29.0)	0.231
Beta-blockers	723 (69.2)	249 (64.3)	474 (72.0)	0.009
Statins	555 (53.1)	199 (51.4)	356 (54.1)	0.401
Diuretics	571 (54.6)	242 (62.5)	329 (50.0)	<0.001
Antiplatelet therapy	256 (24.5)	123 (31.8)	133 (20.2)	<0.001
ACE inhibitors	255 (24.4)	89 (23.0)	166 (25.2)	0.418
Angiotensin II receptor blockers	456 (43.6)	168 (43.4)	288 (43.8)	0.910
Oral antidiabetics/insulin	279 (26.7)	94 (24.3)	185 (28.1)	0.177
CHA_2_DS_2_-VASc, median (IQR)	4 (3–5)	4 (3–5)	4 (2–4)	<0.001
HAS-BLED, median (IQR)	2 (2–3)	3 (2–3)	2 (2–3)	0.001

*ACE inhibitors*, angiotensin-converting-enzyme inhibitors; *COPD/OSAH*, chronic obstructive pulmonary disease/obstructive sleep apnea/hypopnea syndrome; *BMI*, body mass index; *IQR*, interquartile range; *TIA*, transient ischemic attack

CHA2DS2-VASc = congestive heart failure or left ventricular dysfunction (1 point); hypertension (1 point), age ≥75 (2 points) or 65-74 (1 point), diabetes mellitus (1 point), prior stroke/TIA or systemic embolism (2 points), vascular disease (peripheral artery disease, myocardial infarction, aortic plaque) (1 point), sex category (i.e., female sex) (1 point); HAS-BLED = hypertension (1 point), abnormal renal and/or liver function (1 point), prior stroke (1 point), bleeding history or predisposition (1 point), labile INR (1 point), elderly (1 point), drugs or excess alcohol (1 point each)

Regarding the ABC pathway, 97.3% (*n* = 1017) of patients fulfilled “A” criterion at baseline; 85.2% (*n* = 890) fulfilled “B” criterion; and 77.4% (*n* = 809) fulfilled “C” criterion. Overall, 3.0% (32) of patients were adherent to only one criterion, 34.0% (355) were adherent to two criteria and 63.0% (658) were adherent to the three criteria. Thus, 63.0% (658) were categorized as adherent to the ABC pathway at baseline, whereas 37% (387) were considered not adherent.

Although there was no significant difference in age, patients non-adherent to the ABC pathway presented with more prevalent diabetes, heart failure, renal impairment, and vascular disease (both coronary and peripheral artery diseases), as well as with higher CHA_2_DS_2_-VASc and HAS-BLED scores, compared to adherent patients (Table 1).

In terms of OAC therapy, the mean TTR was lower in those non-adherent to the ABC pathway compared to ABC-adherent patients (59.4%±22.3% vs. 63.9%±21.1%; *p*=0.002), and among those non-adherent to the ABC pathway at baseline, a higher proportion presented with a TTR <65% than TTR ≥65% (56.4% vs. 43.6%, *p*=0.023). In addition, 144 (21.9%) patients adherent to the ABC pathway switched to a DOAC during the follow-up, and 67 (17.3%) non-adherent patients did so (*p*=0.195).

### Clinical Outcomes During the Follow-up

During a median follow-up of 2 years, there were 172 (16.5%) all-cause deaths, 261 (25.0%) net clinical outcomes, 164 (15.7%) MACEs, and 110 (10.5%) composite thrombotic/thromboembolic events. Compared to patients non-adherent to the ABC pathway, compliant patients showed significantly lower event rates for the primary outcomes of net clinical outcomes (19.65 [95% CI 16.32–23.46] vs. 11.94 [95% CI 10.04–14.10] per 100 PYs; *p*<0.001) and MACE (11.88 [95% CI 9.32–14.91] vs. 7.75 [95% CI 6.23–9.52] per 100 PYs; *p*=0.006). The primary outcome of all-cause mortality was higher in patients non-adherent to the ABC pathway than that in adherent patients (13.76 [95% CI 11.08–16.90] vs. 6.56 [95% CI 5.21–8.15] per 100 PYs; *p*<0.001) with IRR of 2.10 (95% CI 1.54–2.87) and this was also observed for cardiovascular death (IRR 2.41, 95% CI 1.51–3.89, *p*<0.001).

A detailed comparison of annual event rates and incidence rate ratios according to the ABC pathway groups is shown in Table 2.

**Table 2.** Comparisons of Annual Event Rates and Incidence Rate Ratios for the Different Endpoints Between ABC Pathway Groups

	**ABC pathway non-adherent** ***N*** **= 387**		**ABC pathway adherent** ***N*** **= 658**		***p*-value**	**Incidence rate ratio (95% CI)**
	***N*** **(%)**	Annual event rate (95% CI)*	***N*** **(%)**	Annual event rate (95% CI)*		
**Ischemic stroke**	15 (3.9)	2.35 (1.32–3.88)	24 (3.6)	2.03 (1.30–3.02)	0.656	1.16 (0.56–2.30)
**Ischemic stroke/TIA**	26 (6.7)	4.08 (2.67–5.98)	40 (6.1)	3.39 (2.42–4.61)	0.460	1.20 (0.71–2.02)
**Acute coronary syndrome**	17 (4.4)	2.64 (1.54–4.23)	26 (4.0)	2.17 (1.42–3.19)	0.533	1.21 (0.62–2.32)
**Venous thromboembolism**	5 (1.3)	0.76 (0.25–1.78)	4 (0.6)	0.33 (0.09–0.83)	0.191	2.34 (0.50–11.81)
**Intracranial hemorrhage**	6 (1.6)	0.92 (0.34–2.01)	5 (0.8)	0.42 (0.13–0.98)	0.181	2.20 (0.56–9.12)
**Major bleeding**	24 (6.2)	3.69 (2.36–5.49)	40 (6.1)	2.51 (1.79–3.42)	0.135	1.47 (0.85–2.50)
**CRNMB**	54 (14.0)	8.93 (6.71–11.65)	99 (15.0)	8.80 (7.15–10.71)	0.933	1.01 (0.71–1.43)
**Major bleeding/CRNMB**	76 (19.6)	12.84 (10.12–16.07)	130 (19.8)	11.84 (9.89–14.06)	0.575	1.08 (0.81–1.45)
**All-cause death**	91 (23.5)	13.76 (11.08–16.90)	81 (12.3)	6.56 (5.21–8.15)	<0.001	2.10 (1.54–2.87)
**Cardiovascular death**	44 (11.4)	6.66 (4.84–8.94)	34 (5.2)	2.76 (1.91–3.85)	<0.001	2.41 (1.51–3.89)
**Net clinical outcomes**	122 (31.5)	19.65 (16.32–23.46)	139 (21.1)	11.94 (10.04–14.10)	<0.001	1.65 (1.28–2.11)
**MACE**	74 (19.1)	11.88 (9.32–14.91)	90 (13.7)	7.75 (6.23–9.52)	0.006	1.53 (1.11–2.11)
**Composite thrombotic/thromboembolic events**	42 (10.9)	6.76 (4.87–914)	68 (10.3)	5.83 (4.52–7.39)	0.447	1.16 (0.77–1.73)

*CRNMB*, clinically relevant non-major bleeding; *TIA*, transient ischemic attack; *MACE*, major adverse cardiovascular events; *CI*, confidence interval

*Expressed as the number of events per 100 patients-years

### Univariate and Multivariate Analyses

**Figure 1.**
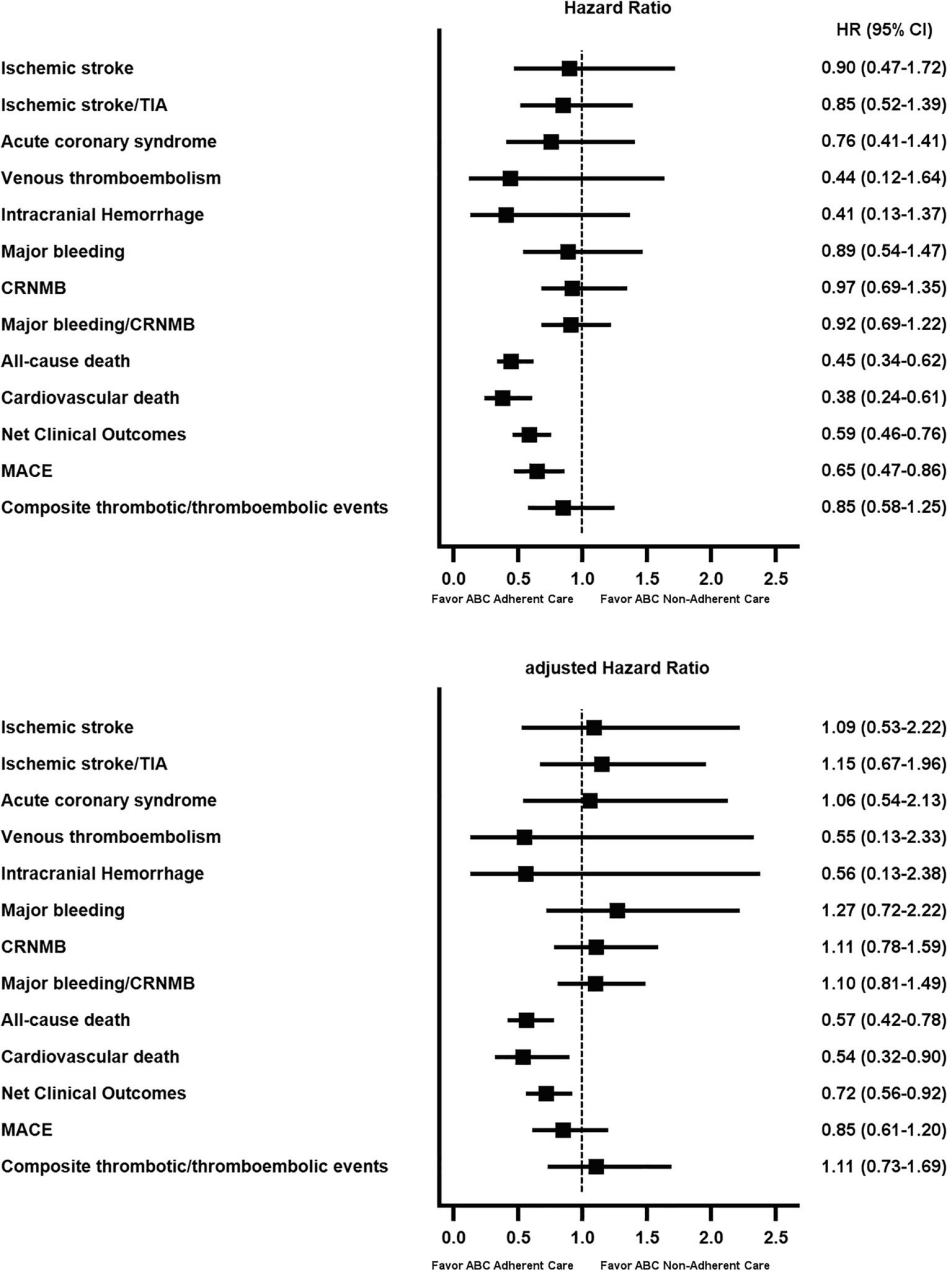
Forest plot of non-adjusted and adjusted hazard ratios for the primary and secondary outcomes according to the ABC pathway adherent care. TIA, transient ischemic attack; CRNMB, clinically relevant non-major bleeding; MACE, major adverse cardiovascular events. *Adjusted hazard ratios by the following variables: age, sex, hypertension, diabetes, ischemic stroke/TIA/SE, vascular disease, heart failure, chronic kidney disease, history of bleeding, alcohol abuse, hepatic disease, and cancer.

Regarding the risk assessment, univariate analyses showed that all risks of outcomes were lower in patients adherent to the ABC pathway, being significant for all-cause mortality, cardiovascular mortality, net clinical outcomes, and MACE (Fig. 1, panel A). After adjusting for several comorbidities, the ABC adherent care favored the reduction of the risk of all-cause mortality (aHR 0.57, 95% CI 0.42–0.78), cardiovascular mortality (aHR 0.54, 95% CI 0.32–0.90), and net clinical outcomes (aHR 0.72, 95% CI 0.56–0.92) (Fig. 1, panel B). Adjusted HRs for the primary outcomes according to the number of ABC pathway criteria fulfilled are shown in Supplementary Figure 1.

**Figure 2.**
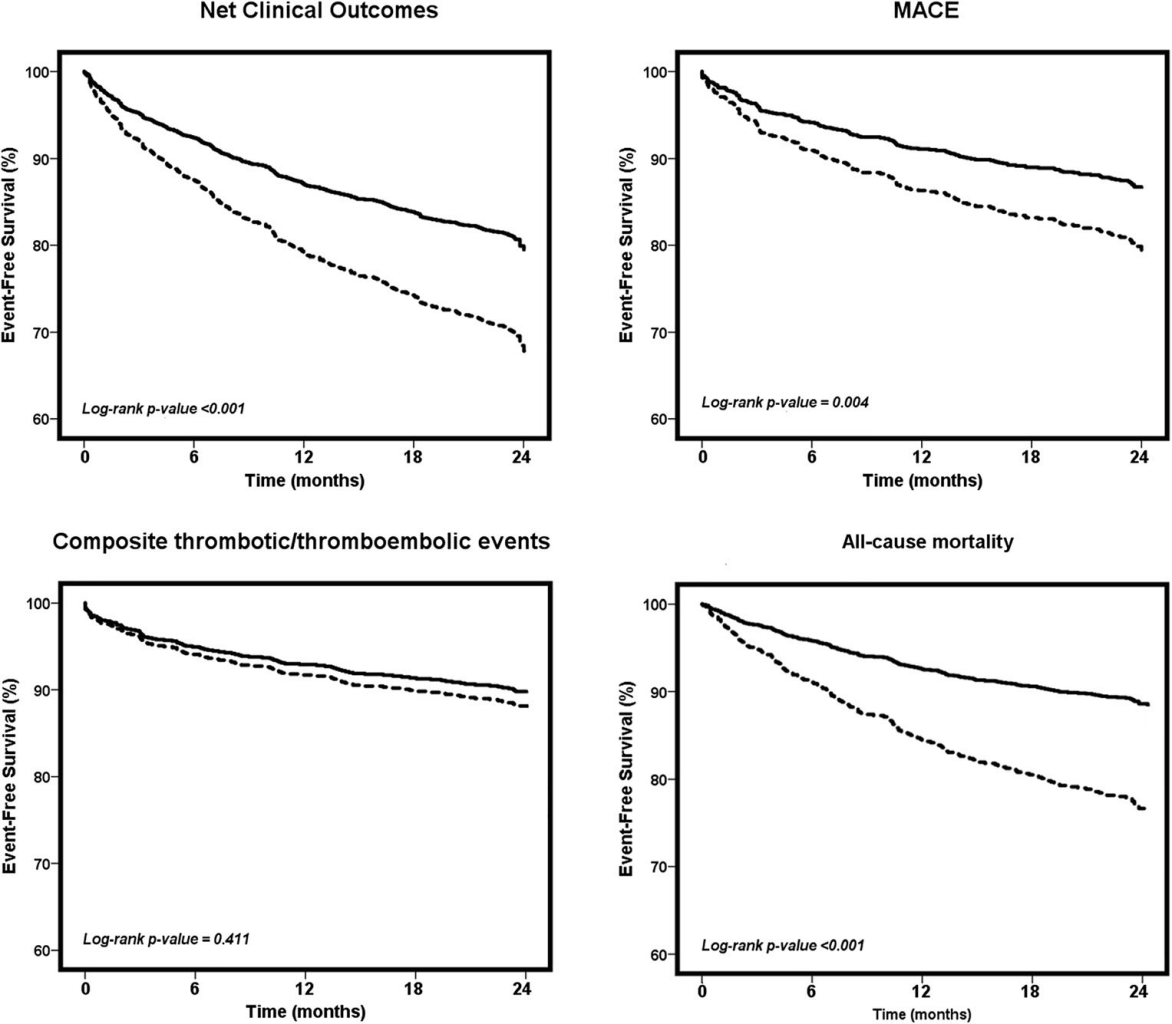
Kaplan-Meier survival curves for the primary outcomes according to the ABC pathway adherent care. Solid line, adherent patients to the ABC pathway. Dashed line, non-adherent patients to the ABC pathway.

Kaplan-Meier analyses showed that event-free survival was generally reduced in non-adherent patients to the ABC pathway as compared to adherent patients. At 2 years, non-adherent patients presented significantly lower survival in terms of net clinical outcomes and all-cause mortality (both log-rank *p*-values <0.001), as well as MACE (log-rank *p*-value = 0.004), but not in terms of composite thrombotic/thromboembolic events (log-rank *p*-value = 0.411) (Fig. 2).

## DISCUSSION

In this prospective cohort study including real-world AF patients, we found that patients adherent to the ABC pathway at baseline had a significantly lower risk of net clinical outcomes, all-cause mortality, and cardiovascular death.

Despite differences in some comorbidities between ABC pathway adherent and non-adherent patients, many patients in the non-adherent group were included in such group not exclusively for having a particular comorbidity but for having the comorbidity not correctly managed. Therefore, the differential point between adherent and non-adherent patients is not the proportion of comorbidities itself but if they were appropriately addressed. Moreover, the risks of net clinical outcomes, all-cause mortality, and cardiovascular death are presented after adjustment for specific comorbidities, including those that were significantly different between groups. This novel approach, not so focused on the disease itself but more on the patient, could be useful in our population, and represents a paradigm shift in relation to the management of patients with AF, which has tended to focus on individual strategies (e.g., stroke prevention, or rate vs. rhythm control).

Although this is the first prospective study investigating the usefulness of the ABC pathway in our national context, overall, our results reinforce previous retrospective observations, despite that the proportion of adherent patients to the ABC pathway was higher in our cohort compared to prior studies.

Since its first description in 2017^1^, the ABC pathway has been tested in different regions of the world showing that better adherence improves patient outcomes. For example, Proietti et al. first performed a post hoc analysis in 3169 AF patients from the AFFIRM trial. The authors found that patients managed according to the ABC pathway had lower risk of all-cause death (aHR 0.35; 95% CI 0.17–0.75), composite outcome of stroke/major bleeding/cardiovascular death (aHR 0.35; 95% CI 0.18–0.68), and first hospitalization (aHR 0.65; 95% CI 0.53–0.80)^6^. Subsequently, Pastori et al. investigated the impact of implementing the ABC pathway on cardiovascular events in consecutive real-world AF patients, showing significantly lower risks of the composite outcome of fatal/nonfatal ischemic stroke and myocardial infarction (MI), TIA, cardiac revascularization, and cardiovascular death (aHR, 0.44, 95% CI 0.24–0.80)^9^. The ABC pathway is particularly beneficial in men and in AF patients with a 2MACE score ≥3, since these presented a higher risk of MACE^10^.

Another study using a nationwide population cohort demonstrated that patients who complied with the ABC pathway presented a significantly lower risk of all-cause death (aHR 0.82; 95% CI 0.78–0.86) and the composite outcome of all-cause mortality, ischemic stroke, major bleeding, and myocardial infarction (aHR 0.86; 95% CI 0.83–0.89). Importantly, the risk of all-cause death and composite outcome were progressively lowered with the increasing numbers of ABC pathway criteria fulfilled^8^. In a retrospective analysis from the Gulf SAFE registry, an observational study including AF patients from the Middle East, patients adherent to the ABC shower significantly lower risk of the composite outcome of ischemic stroke/systemic embolism, all-cause mortality, and cardiovascular hospitalization (aHR 0.53; 95% CI 0.36–0.8) and all-cause mortality alone (aHR 0.46; 95% CI 0.25–0.86)^19^. Similarly, ABC pathway compliance showed an independent association with reduction of all-cause death and the composite of all-cause death, ischemic stroke, and intracranial hemorrhage in a large cohort of Chinese AF patients^20^.

The ABC pathway has also been introduced using mobile health (mHealth) technology for AF care. For example, in a cluster randomized trial that randomized AF patients to usual care or to an integrated care approach based on a mobile AF Application (mAFA) incorporating the ABC pathway, the mAFA intervention decreased the risk of the composite outcome of “ischemic stroke/systemic thromboembolism, death, and rehospitalization” (HR 0.39; 95% CI 0.22–0.67) and rehospitalization (HR 0.32; 95% CI 0.17–0.60)^11^. Finally, the impact on outcomes of an ABC adherent management has been evaluated in the ESC-EHRA EURObservational Research Programme in AF General Long-Term Registry. After adjusting for several confounding factors, again the ABC adherent care showed an association with a lower risk of the composite of any thromboembolism/acute coronary syndrome/cardiovascular death (HR 0.59, 95% CI 0.44–0.79), cardiovascular death (HR 0.52, 95% CI 0.35–0.78), and all-cause death (HR 0.57, 95% CI 0.43–0.78)^21^. Indeed, the clinical usefulness of the ABC pathway is evident even in high-risk populations such as frailty, diabetes, or those with multiple comorbidities, polypharmacy, or prior hospitalizations^7,22–24^; and application of the ABC pathway may even reduce healthcare costs related to cardiovascular events^25^.

However, most of the studies regarding the ABC pathway were performed in DOAC-treated AF populations whereas VKAs are still widely used in several countries globally. In such patients, quality of anticoagulation and appropriate TTR is central. Recently, we have reported that the ABC pathway adherent patients had better TTR, and more ABC criteria fulfilled increased the probability of achieving good TTR^26^. Thus, ABC pathway management is not only associated with a lower number of clinical outcomes but also results in better anticoagulation control, which in turn is associated with a better prognosis.

The above evidence reinforces the hypothesis that AF management requires a holistic approach. In fact, not only do the 2020 ESC guidelines for the management of AF^2^, 2021 Asia Pacific Heart Rhythm Society guidelines^27^, the 2018 CHEST guidelines^28^, and the Korean Heart Rhythm Society guidelines^29^ suggest focusing on such approach, but the 2020 Canadian Cardiovascular Society/Canadian Heart Rhythm Society guidelines also suggest a structured, integrated, multidisciplinary, patient-focused approach for patients with AF^30^.

Beyond stroke prevention, symptom management and control of cardiovascular risk factors are equally needed in AF. Indeed, multi-morbidity is common among AF patients and contributes to worse clinical outcomes and quality of life^31^. Therefore, the importance and necessity of modifying cardiovascular risk factors is crucial, and this has demonstrated to decrease disease burden and progression^32^. Integrated care, by putting the patient into the center and including other healthcare professionals, aids this objective^18,33^. Previous specialized AF clinics and nurse-led AF clinics have already included this approach with promising results^34–36^.

### Limitations

The main limitation of the study lies in its observational nature, with a Caucasian-based population and single-center design. Another potential limitation is patient selection since we included only patients starting OAC therapy with VKA for the first time. Previous studies have shown that the initial period of OAC is characterized by an increased risk of adverse events, particularly bleeding ones, and during the first 3 months of VKA therapy, which may have some influence on the results^37–39^. However, our dataset was collected prospectively, under a careful follow-up. Thus, all events (even very early ones) were recorded. Importantly, patients lost to follow-up were excluded for the present analysis.

We must recognize a potential drug bias because inter-physician changes of drugs prescription might have an impact on the ABC adherent care and therefore on clinical outcomes. However, we specifically tested the impact of baseline adherence to the ABC pathway on mid-term outcomes. Although several physicians could be responsible for pharmacological therapies prescribed to patients, the same hematologist from an anticoagulation clinic was in charge of all patients of the study, thus avoiding the bias of different criteria from different hematologists regarding anticoagulation therapy. Notwithstanding, the management may differ in other settings, and the generalization to other centers with less intense follow-up or including mainly patients under DOACs requires further investigation. Finally, we did not revaluate adherence to the ABC pathway during the 2 years follow-up, which we recognize could have some impact on clinical outcomes.

## CONCLUSIONS

In a large prospective cohort of real-world AF patients starting VKA therapy, adherence to ABC pathway management was demonstrated to significantly reduce the risk of net clinical outcomes, all-cause mortality, and cardiovascular death at 2 years. A structured, holistic, and integrated care approach, based on the ABC pathway, is advantageous for AF management, improving patient outcomes.

## Supplementary information

ESM 1 (DOCX 177 kb)
