# Supplementary material for: The Atrial Fibrillation Better Care (ABC) Pathway and Clinical Outcomes in Patients with Atrial Fibrillation: the Prospective Murcia AF Project Phase II Cohort
Source: J Gen Intern Med. 2022 Apr 11;38(2):315–23. doi: 10.1007/s11606-022-07567-5 (PMC9905403; doi:10.1007/s11606-022-07567-5)
Supplement: Supplementary file 1 — (DOCX 177 kb) [file 11606_2022_7567_MOESM1_ESM.docx]

**Supplementary Material**

**Supplementary Figure 1.** Forest plot of hazard ratios for the primary outcomes according to the number of the ABC pathway criteria fulfilled.

**
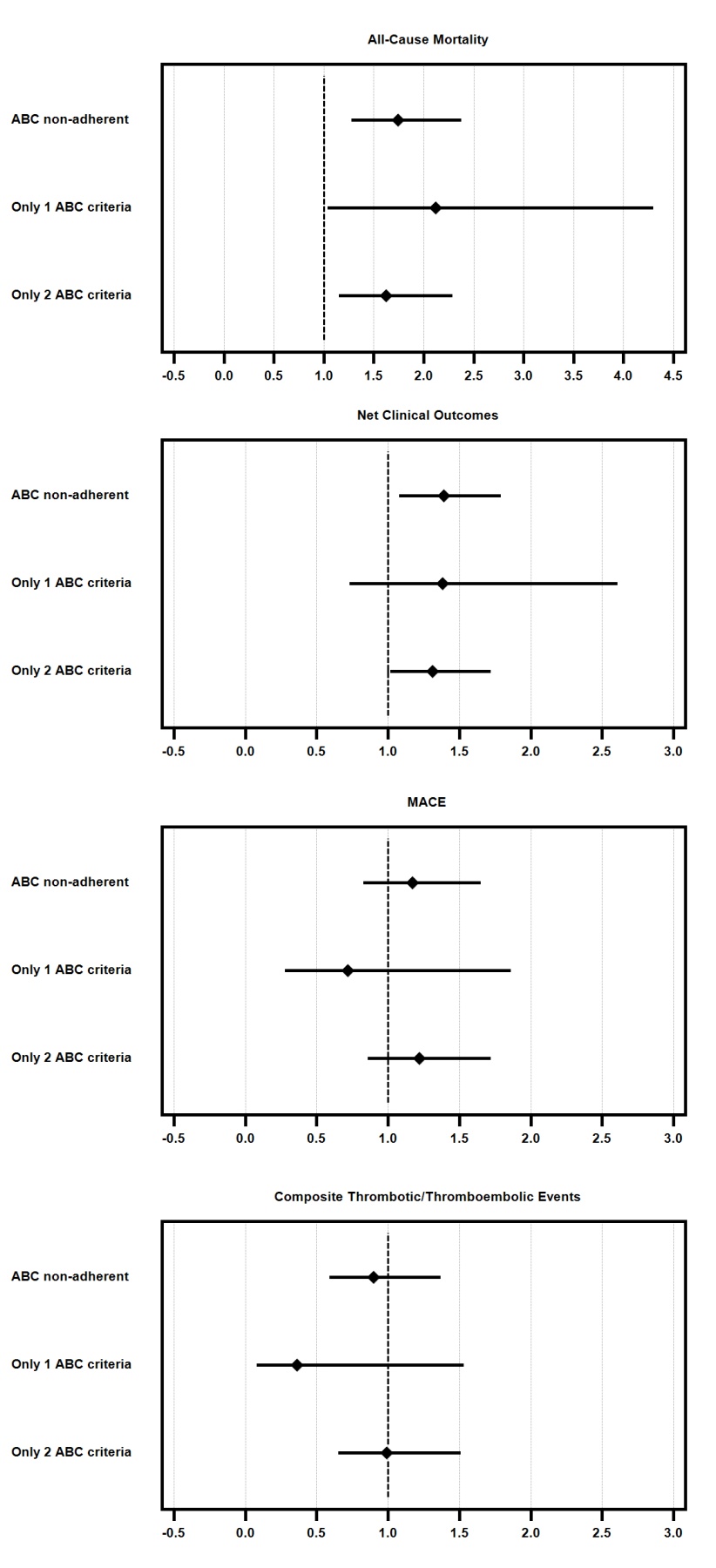
**
